# The effect of endurance training on non‐alcoholic fatty liver disease in mice

**DOI:** 10.14814/phy2.14926

**Published:** 2021-08-02

**Authors:** Luma Melo, Merve Bilici, Amit Hagar, James E. Klaunig

**Affiliations:** ^1^ Laboratory of Investigative Toxicology and Pathology Department of Environmental and Occupational Health Indiana School of Public Health Indiana University Bloomington IN USA; ^2^ History and Philosophy of Science and Medicine Department Indiana University Bloomington IN USA; ^3^ Intelligent Systems Engineering Department Indiana University Bloomington IN USA

**Keywords:** endurance exercise, exercise, liver, metabolism, NASH

## Abstract

Chronic endurance exercise is a therapeutic strategy in the treatment of non‐alcoholic fatty liver disease (NAFLD). Metabolic, cardiorespiratory, and endocrine pathways targeted by chronic endurance exercise have been identified; however, the specific cellular and molecular pathways modified by exercise in the steatotic liver remain unresolved. In this study, we show hepatic gene expression, and the structure, characteristics, and clinical differences between sedentary and exercised mice, by an endurance exercise model with wheels with a controlled velocity that allows for the quantification of a human‐relevant endurance “dosage,” after exposure to regular and high‐fat diet. Chronic exercise modified the transcription of hepatic genes related to liver nuclear receptors, cell growth, fibrosis, inflammation, and oxidative stress, and decreased the amount of lipid accumulation in the liver. Moreover, the combination of endurance training with the change in diet differentially modified the genetic expression of the biomarkers relative to the separate interventions. Even though exercise by itself showed counteract NAFLD development, the combined intervention was sufficient to convert the structure and clinical aspects of the liver from steatotic to healthy. Given our findings, the combination of endurance exercise and change in diet should be considered a therapeutic option for NASH.

## Graphical abstract

Chronic exercise ameliorated fibrosis, inflammation, and oxidative stress, through the transcription of hepatic genes related to liver nuclear receptors, cell growth, fibrosis, inflammation, and oxidative stress. Chronic exercise decreased the amount of high‐fat diet‐induced lipid accumulation in the liver. The combination of endurance exercise and change of diet should be considered a therapeutic option for NASH.

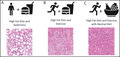

## 1 INTRODUCTION

Non‐alcoholic fatty liver disease (NAFLD) affects almost one‐third of the Western adult population (Adams et al., 2005) and it is the most common etiology of Chronic Liver Disease globally, affecting ~25% of the population (Younossi et al., 2016). The spectrum of liver damage related to NAFLD ranges from simple steatosis to cirrhosis and ultimately liver cancer, with the presentation of inflammation and hepatocellular injury, with or without fibrosis (Chalasani et al., 2018). The development of NAFLD is associated with metabolic syndrome, since patients with metabolic syndrome such as obesity, dyslipidemia, insulin resistance, and hypertension (Anstee & Goldin, 2006; Pimpin et al., 2018) tend to have a higher risk of developing NAFLD (Paschos & Paletas, 2009).

Diet, lipolysis, and de novo lipogenesis are the three major suppliers of free fatty acids in hepatocytes (Donnelly et al., 2005). A high‐fat diet may increase the amount of fatty acids transported into the circulation and delivered to the liver, promoting steatosis in the liver by increasing lipid uptake. Hepatocytes absorb fatty acids proportional to their concentration in the blood. Moreover, a dietary supply of excessive carbohydrates was shown to activate genes related to de novo fatty acid synthesis (Dentin et al., 2006; Postic & Girard, 2008), impairment of insulin‐mediated suppression, and fatty acid catabolism in hepatocytes (Anstee & Goldin, 2006).

Given the etiology of NAFLD, its progression is generally seen in sedentary patients with no physical activity in their routine (Croci et al., 2019). In a recent study, moderate‐intensity exercise (nearly 150 min per week) was associated with decreasing hepatic steatosis and liver inflammation in patients with NAFLD (Thorp & Stine, 2020). In mice fed with high‐fat diet, regular exercise resulted in a decrease in the size of hepatic lipid droplets, lower triglyceride concentration, and reduced liver damage (Pino‐de la Fuente et al., 2019).

Some of the pharmaceutical strategies proposed to treat NAFLD include antioxidants, anti‐obesity, and mitochondrial‐targeted drugs; however, their therapeutic efficiency levels are low and the U.S. Food and Drug Administration (FDA) has not yet approved any of them (Stevanović et al., 2020). Given the limited effects of pharmacological intervention, regular exercise, mainly endurance, has been suggested as the most effective current strategy to overcome the liver pathology associated with fatty liver disease (Stevanović et al., 2020). Regular endurance exercise is believed to be a protective approach measure against metabolic disorders associated with high‐fat diet, and increased physical activity along with the restriction of fat and calorie intake is considered the best way to maintain healthy liver conditions (Johnson & George, 2010).

In this study, we aimed to exam the effect of exercise on clinical markers of NAFLD in a mouse liver by human‐relevant rodent models for NAFLD and exercise. We hypothesized that exercise will be able to ameliorate the NAFLD onset; however, the only exercise along with diet change intervention will have the potential for reverting the fatty liver to a healthy liver. Liver gene expression of specific biomarkers for liver diseases and histology slides of mice exposed to control diet and high‐fat diet for 12 weeks and then gradually trained with a stressless, controllable, and quantifiable forced running protocol for 8 weeks were analyzed. We were able to detect that the exercise reduces the liver lipid content, and combined with a change in diet, reverses HFD‐induced steatosis. We further found that exercise ameliorated HFD induced hepatic fibrosis and collagen content and activates hepatic nuclear receptors. We hope this study consolidates the benefits of exercise itself in non‐alcoholic liver disease prevention, progression, and the combination with change in diet.

## 2 MATERIALS AND METHODS

### 2.1 Laboratory animals and husbandry

**FIGURE 1:**
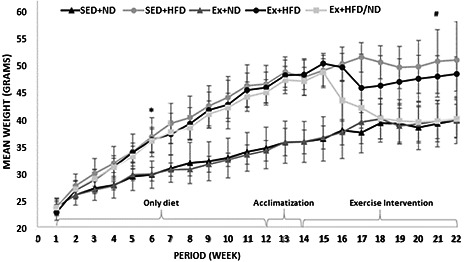
Effects of diet and exercise on weekly measured body weight. High‐fat diet affected body weights and exercise alone does not affect body weight significantly. However, the change in diet with exercise reversed body weight and liver weight. Values represent mean ± SD (N = 4‐5/group). One‐way ANOVA (Tukey's range post hoc test) was used for testing statistical significance. * indicates a significance grreater than p<0.05. Significantly different compared to the corresponding control sedentary. P‐values are available as Supplementary Data S4

**FIGURE 2:**
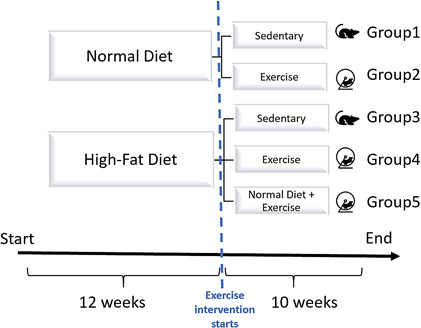
Treatment protocol for 22 weeks. Fifty‐five to six‐week‐old male C57BL/6 5‐week‐old mice were divided randomly into five groups (10 mice in each group). Group 1 had access to control diet (ND) and did not exercise (sedentary), group 2 also had access to control diet and were endurance‐trained, group 3 had access to high‐fat diet (HFD) and stayed sedentary for the entire study, group 4 were also exposed to HFD and was endurance‐trained, and group 5 had access to high‐fat diet (HFD) for 12 weeks, and then changed the diet from HFD to control diet while endurance‐trained

Five‐ to six‐week‐old male C57BL/6 mice were purchased from The Jackson Laboratories (Maine, USA) were housed at the Indiana University Bloomington animal facility five per cage in ventilated cages at a controlled temperature of 22°C in a 12:12‐h light–dark cycle, and relative humidity of 55±10. All mice had ad libitum access to food and water. Individual body weights of mice were measured weekly (Figure 1). Food intake was measured by weighing the food left within the cage on two consecutive days once a week. After a 7‐day acclimation period, all mice were randomly divided into five groups (Figure 2): group 1 (SED + ND) had access to control diet (ND) (13.5% calories from fat; TD.120336 Harlan Teklad) and did not exercise (sedentary), group 2 (EX + ND) also had access to control diet and were endurance‐trained , group 3 (SED + HFD) had access to high‐fat diet (HFD) (42% calories from fat; TD.09682 Harlan Teklad) and stayed sedentary for the entire study, group 4 (EX + HFD) was also exposed to HFD and was endurance‐trained, and group 5 had access to high‐fat diet (HFD) for 12 weeks, and then changed the diet from HFD to control diet while endurance‐trained (Table 1). The exercise training protocol followed that previously described (Hagar et al., 2019; Melo et al., 2021). Briefly, during the first 2 weeks, mice were acclimatized to the training wheels. In the following 8 weeks, mice slowly increased their training time as well as the maximum target velocity, so that on the final week they run 26 min/day with a maximum velocity of 12 m/min for the last 2 min (this velocity was not imposed on the mice as an a priori target but was discovered through repeated training as their highest tolerance; it corresponds on average to 70% of their maximum VO_2_ capacity (Karlsen et al., 2017)). Upon any sign of distress within the mice, the velocity was reduced to allow the animal to run naturally again. Mice from the sedentary groups (groups 1 and 3) were placed inside the wheels but did not run for the same amount of time that the other groups would run. Mice were humanely euthanized and liver tissues were collected. Part of the liver was fixed in 10% neutral‐buffered formalin for 24 hours and transferred into 70% ethanol for histology analysis, and the rest was snap‐frozen for biomedical and gene expression analysis.

**TABLE 1:** Termination body weights, liver weights, and relative liver weights. High‐fat diet affected body and liver weights and change in diet with exercise reversed body weight and liver weight to a value comparable to group 1

Group	Group	Body Weight(g)	Liver Weight(g)	Relative Liver Weight (%)
1	Sed + ND	37.92 ± 3.87	1.79 ± 0.57	4.66 ± 0.97
2	Sed + HFD	49.27 ± 5.77^a^	5.09 ± 1.09^a^	10.59 ± 3.5^a^
3	Ex + ND	37.33 ± 3.10	1.72 ± 0.66	4.54 ± 1.36
4	Ex + HFD	45.96 ± 2.95^b^	3.35 ± 0.88^b^	7.23 ± 1.50^b^
5	Ex + HFD/ND	37.57 ± 3.17^c^	1.80 ± 0.57^c^	4.70 ± 1.16^c^

Data are displayed as Mean ± SD. One‐way ANOVA or t‐test was used for testing statistical significance (*p* < 0.05).

*p*‐values are available as Supplementary Data S4 and S6. Effect size f equal to 1.686548.

^a^ Significantly different compared to SED + ND.

^b^ Significantly different compared to EX + ND.

^c^ Significantly different compared to EX + HFD.

### 2.2 Histology and digital image analyses

Previously fixed liver tissues were embedded in paraffin, sectioned, and stained with hematoxylin and eosin (H&E) for histopathologic examination. Additional sections were stained with Masson's trichrome for the evaluation of collagen deposition and Sirius Red stain for hepatic fibrosis detection. Stained slides were scanned by the ScanScope CS slide scanning system at ×20 magnification. Liver lipid droplet size and fibrosis quantification were quantitated using Image J. More detail can be found in Supplementary Data S2.

### 2.3 Real‐time PCR analysis

Hepatic RNA was isolated using the RNeasy Plus Mini Kit (Qiagen, Germany) following instructions from the manufacturer. The quality and quantity of the isolated RNA were determined using a NanoDrop 2000 (Thermo Scientific, USA), and the integrity was examined using a denaturing agarose gel (1.2%) stained with ethidium bromide. Samples with high quality and quantity of RNA were further sourced for complementary DNA synthesis with Superscript III First‐Strand Synthesis System (Life Technologies, USA) as manufacturer's guidelines instruct. Liver nuclear receptor, cell growth, fibrosis, inflammation, and oxidative stress‐related genes were assessed using quantitative real‐time PCR (qPCR). The gene primer sequences for 56 primers were constructed and are listed in Supplementary Data S1. The SYBR Green detection method was used along with β2m as housekeeping control for each PCR reaction.

### 2.4 Statistical analysis

All data are expressed as mean ± SD. Statistical difference, significantly different when *p* < 0.05, was determined using either student t‐test for two groups or ANOVA when comparing three or more groups. Post hoc analysis was conducted to investigate changes between groups, if a statistically significant ANOVA result was identified. P‐values are individually available as Supplementary Data S4, S5, and S6. The fold change of each RNA sequence examined was calculated using the 2^−ΔΔCt^ method according to Livak and Schmittgen (2001).

## 3 RESULTS

### 3.1 Effects of exercise and HFD on body and liver weights

As previously shown by our group (Li et al., 2019), mice in the high‐fat diet groups (groups 3, 4, and 5) gained weight significantly (*p* < 0.05) after week 6 (Figure 1) when compared to the normal diet groups (groups 1 and 2). Final liver weight measurements showed the same significant difference between the high fat and diet and normal diet (Table 1). For mice fed with control diet (ND) (groups 1 and 2) and mice fed with high‐fat diet (HFD) (groups 3 and 4), exercise did not affect the terminal body weight or terminal liver weight (Table 1) significantly (*p* < 0.05). However, the group in which there was a change in diet along with exercise (group 5), the terminal body weight and liver weight were brought down to levels nearly equal to the control diet groups (groups 1 and 2). The change in weekly body weight was able to be seen after week 16. Thus, after 4 weeks of feeding a normal diet with the addition of exercise, the exercise was sufficient to reverse the weekly body weight gains seen after 12 weeks of HFD. A similar effect was seen in final liver weight: the change in diet combined with exercise was sufficient to reverse the gain in liver weight compared to the HFD groups.

### 3.2 Exercise reduces the size of liver fat droplets, and combined with a change in diet, reverses HFD‐induced steatosis

**FIGURE 3:**
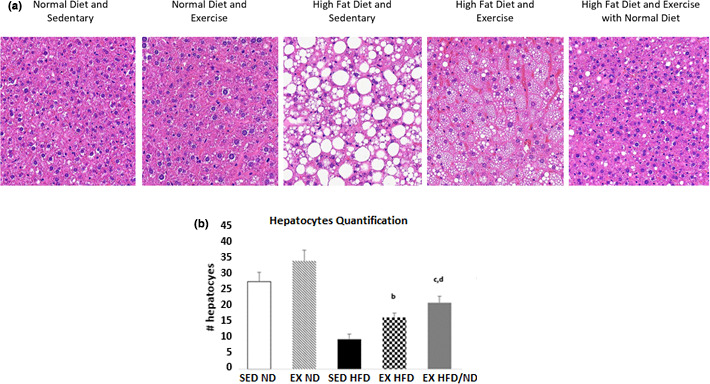
(a) Liver histology examination of lipid accumulation. Exercise changed lipid vesicles from macrovesicular to microvesicular form in the HFD groups. Change in diet along with exercise reversed the lipid accumulation seen by both groups with control diet histopathology slides. (b) Hepatocyte quantification performed with the use of Image J. Values expressed as mean ±SD. *p* < 0.05 is considered significant. a, significantly different from ND SED; b, significantly different from HFD SED; c, significantly different from ND EX; d, significantly different from HFD EX

The analysis of histopathologic changes in the liver showed that feeding of high fat diet (groups 2, 4, and 5) produced and increase in lipid (macrovesicular steatosis) in the liver (Figure 3a). In the exercise‐trained groups (groups 2, 4, and 5), the training was sufficient to reduce the HFD induced hepatic steatosis seen in the HFD sedentary groups (group 3). In addition, the size of hepatic lipid droplets, as well as their form (change from macrovesicular to microvesicular form), was significantly (*p* < 0.05) reduced in group 3 compared to group 4 (Figure 3a). The microvesicular pattern was predominant in the centrilobular (zone 3) and middle zone portions of the liver lobules. This pattern in this study in the mouse was consistent with that reported in human NASH in which both micro‐ and macrovesicular steatosis are predominant in the centrilobular area of the lobule (Takahashi & Fukusato, 2014).

**FIGURE 4:**
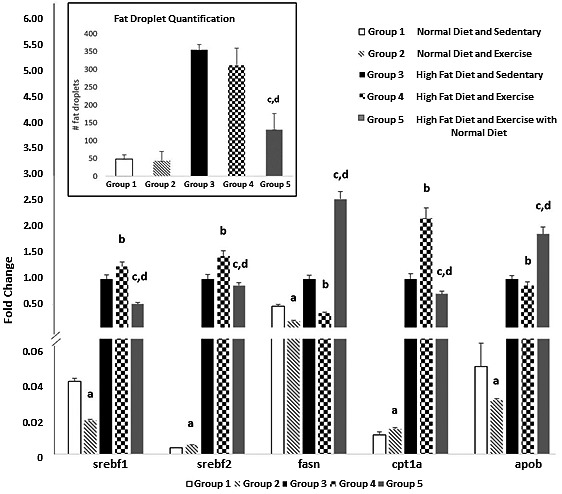
Gene expression of genes related to fatty acid metabolism and fat droplet quantification of histology analysis using Image J. Values expressed as mean ±SD. *p* < 0.05 is considered significant. a significantly different from ND SED; b significantly different from HFD SED; c significantly different from ND EX; d significantly different from HFD EX. There was no significant difference in lipid droplet quantification in the exercised mice and control sedentary mice, however, group 5’s fat accumulation was significantly decreased from group 4 and significantly increased from group 2

Liver lipid droplet size was quantified (Figure 4) and there was no significant difference in lipid droplet quantification between HFD groups 3 and 4; however, group 5 (change in diet with exercise) was significantly (*p* < 0.05) lower when compared to group 4 (endurance training and HFD). The lipid accumulation seen by histopathology was significantly (*p* < 0.05) reduced in group 5 and was similar to groups 1 and 2 (both groups with control diet).

The expression of genes related to fatty acid metabolism is shown in Figure 4. Expression of Sterol Regulatory Element Binding Transcription Factor 1 and 2 (SREBF1and SREBF2), involved in cholesterol synthesis, and Carnitine Palmitoyltransferase 1A (CPTIA), involved in lipid transport, increased in the livers of mice given HFD (groups 3, 4, and 5. The exercise (group 4) produced an apparent additive effect on the expression of these genes (Figure 4). In contrast, removal of the high‐fat diet combined with exercise (group 5) decreased the expression of SREBF1, SREBF2, and CPTIA compared to that seen in group 4. Both Fatty Acid Synthase (FASN), involved fatty acid synthase, and Apolipoprotein B (APOB), involved in lipid transport, had similar behavior. Exercise alone decreased the expression of both genes in accordance to Van der Windt et al., (2018); however, combining with a change in diet (group 5) acted in an opposite direction, enhancing the expression.

### 3.3 Exercise ameliorated HFD induced hepatic fibrosis and collagen

**FIGURE 5:**
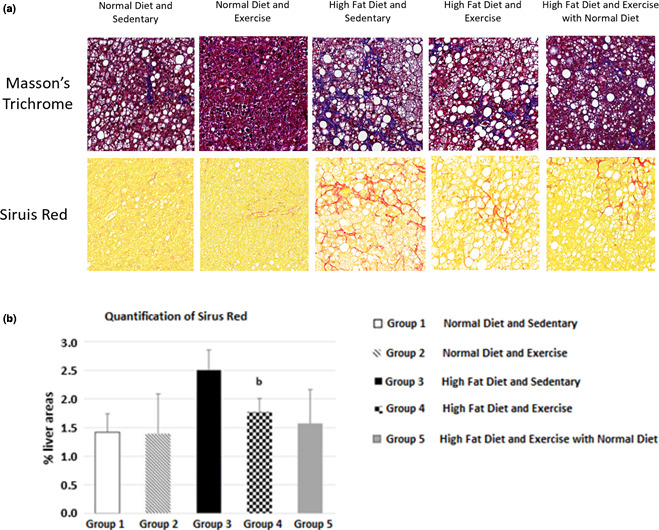
(a) Sirius red and Masson's Trichrome staining of liver and (b) quantification of Sirius red staining using Image J. HFD increased the collagen and fibrotic deposition in the liver and exercise reduced it. Values expressed as mean ± SD. *P* < 0.05 is considered significant. a significantly different from ND SED; b significantly different from HFD SED; c significantly different from ND EX; d, significantly different from HFD EX. The significant difference between two quantifications gives information about diet and exercise‐related histological changes in the animal livers

**FIGURE 6:**
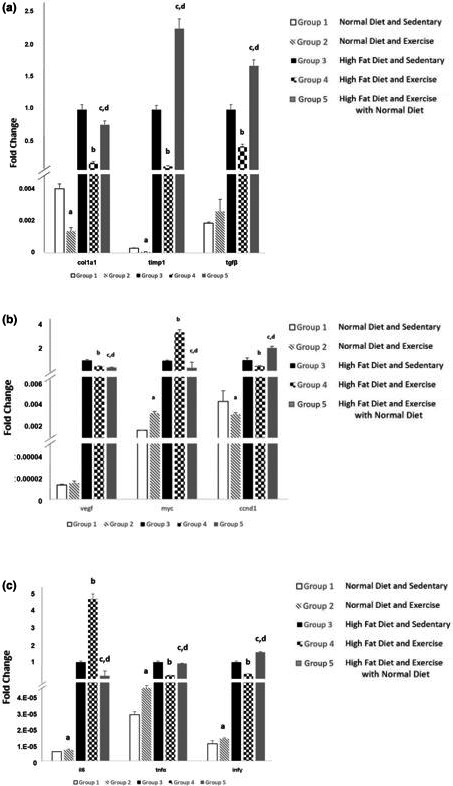
*(*a) Fibrogenic gene expressed. Genes were significantly increased in HFD groups and downregulated after exercise in the HFD groups. (b) Cell proliferation gene expression. MYC was significantly upregulated with exercise in both control and HFD groups, and the change in diet combined with gradual endurance training significantly decreased its expression. VEGF and CCND1 expression were increased in HF groups but decreased significantly with exercise. (c) Inflammation gene expression. The upregulation of these genes was seen with HFD and suggests an increase in hepatic stellate cell proliferation. Values expressed as mean ±SD. *p* < 0.05 is considered significant. a significantly different from ND SED; b significantly different from HFD SED; c significantly different from ND EX; d, significantly different from HFD EX

Histopathologic evaluation showed that high‐fat diet increased both the collagen deposition and fibrosis in the liver (groups 3, 4, and 5) when compared to mice fed with control diet (groups 1 and 2) (Figure 5a). These images show different cross‐sections, the ones shown in Figure 3a. Quantification of Sirius red staining showed an increase with HFD, and a significant 30% decrease of fibrosis when exercise was induced in the high‐fat diet‐fed mice (group 4) when compared to sedentary high‐fat diet mice (group 3) (Figure 5b). Examination of gene expression of fibrotic and collagen correlated with the histopathology results above. Collagen Type I Alpha 1 Chain (COL1A1), TIMP Metallopeptidase Inhibitor 1 (TIMP1), and Transforming Growth Factor Beta (TGF‐β), fibrogenic mRNA expression were significantly (*p* < 0.05) increased in HFD groups (groups 3, 4, and 5) (Figure 6a). These genes appeared to be downregulated after exercise in the high‐fat diet groups, and both COL1A1 and TIMP1 genes were significantly (*p* < 0.05) downregulated after exercise in the control diet groups.

### 3.4 Effects of exercise on HFD‐induced hepatic cell proliferation

Hepatocytes quantification showed a significant decline within the HFD groups, however, exercise was sufficient to significantly (*p* < 0.05) reverse it when compared to the sedentary counterpart (Figure 3b). Change in diet combined with endurance training group (group 5) increased the number of hepatocytes when compared to endurance‐trained HFD fed mice (group 4) and decreased when compared to endurance‐trained control diet group (group 2). The gene expression of cell proliferation and cycle genes Cyclin D1 (CCND1), Vascular Endothelial Growth Factor (VEGF), and MYC was measured to further investigate cell growth (Figure 6b). MYC expression was previously shown to be upregulated with exercise in male C57BL/6 mice liver (Melo et al., 2021), and was also significantly (*p* < 0.05) upregulated in this study with exercise in both control and HFD groups; nevertheless, the change in diet combined with exercise (group 5) significantly (*p* < 0.05) decreased MYC’s expression. VEGF and CCND1 expressions were increased in HF groups but then decreased significantly (*p* < 0.05) with exercise. For group 5 (change in diet combined with endurance training), VEGF was decreased, but CCND1 increased. An increase in CCND1 expression was previously shown to be involved in tissue regeneration, suggesting exercise and change in diet as a better tissue regenerator (Brett et al., 2020).

### 3.5 Exercise reduced HFD hepatic inflammation and oxidative stress

No significant signs of inflammation were seen in the liver histology analyses in all groups, and the hepatic inflammatory response of diet and exercise was further evaluated by the gene expression of Interleukin 6 (IL6), Tumor Necrosis Factor α (TNFα), and Interferon Gamma (IFNγ) (Figure 6c). The upregulation of these genes suggests an increase in hepatic stellate cell proliferation after high‐fat diet exposure and exercise (Van der Windt et al., 2018). In fact, IL6 expression increased significantly (*p* < 0.05) with exercise for both control and HFD. Exercise alone increased TNFα’s expression when compared to control in the ND groups. The effect of exercise by itself on the TNFα levels has been quite controversial (Salamat et al., 2016), as some studies agree that exercise increases the levels of TNFα (Babraj et al., 2009; Ferreira et al., 2009), and others go on the opposite way showing reduction in inflammatory cytokines (Prestes et al., 2009; Silverman et al., 2009). There is still the need for a more in‐depth analysis of different exercise training methods on the modulation of TNFα.

The TNFα, and IFNγ and IL6 expressions, moreover, decreased in the combined intervention (change in diet plus exercise, group 5) when compared to inducing endurance training yet keeping HFD (group 4), suggesting that the combination of change in diet and training decreases hepatic stellate cell recruitment.

**FIGURE 7:**
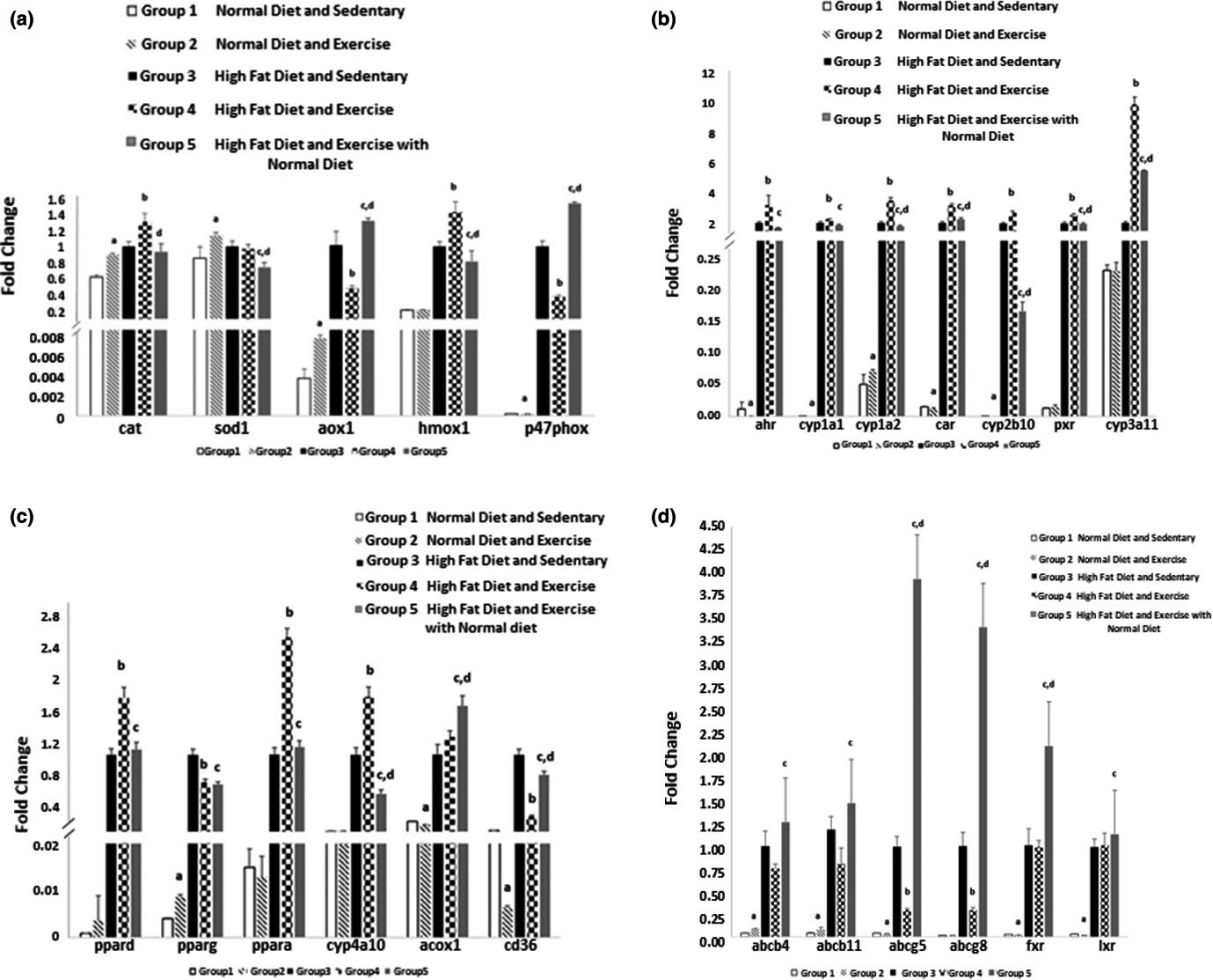
(a) Gene expression of genes related to oxidative stress. AOX1, SOD, HMOX1, and CAT were upregulated with exercise and normal diet when compared to the sedentary and normal diet groups. Change in diet combined with exercise upregulated AOX 1 and P47PHOX, and down‐regulated CAT, SOD1, and HMOX 1 when compared to the trained group which kept its HFD. (b) Gene expression of genes related to Ahr, CAR, and PXR activation. HFD increases all gene expressions and exercise combined with HFD significantly enhanced its expression when compared to the sedentary HFD group. (c) Gene expression of genes related to PPARα, PPARγ, PPARδ activation. PPPARα, PPARδ, and CYP4A10 were significantly enhanced by HFD and exercise along with HFD compared to groups 1 and 2. (d) Gene expression of genes related to LXR and FXR activation. All gene expressions increased with HFD, and exercise had an additive effect on group 5’s expressions. Values expressed as mean ±SD. *p* < 0.05 is considered significant. a significantly different from ND SED; b significantly different from HFD SED; c significantly different from ND EX; d, significantly different from HFD EX

From the oxidative stress‐related genes, adehyde oxidase 1 (AOX1), together with superoxide dismutase (SOD) and catalase (CAT), two of the major antioxidant enzymes, were upregulated with exercise and normal diet when compared to the sedentary and normal diet groups (Figure 7a). Both CAT and heme oxygenase 1 (HMOX1), considered one of the most sensitive and reliable indicators of oxidative stress, were also upregulated with exercise and HFD when compared to sedentary and HFD. HMOX1 is important in the defense against oxidative stress (Chang et al., 2015), and higher HMOX1 activity correlated with less frequent and less severe NAFLD (Severson et al., 2016).

Moreover, the expression of AOX 1 and neutrophil cytosolic factor 1 (P47PHOX), a crucial subunit of NADPH oxidase, decreased in the HFD groups when comparing endurance‐trained to sedentary. Change in diet combined with exercise upregulated AOX 1 and P47PHOX, and downregulated CAT, SOD1, and HMOX 1 when compared to the trained group which kept its HFD. Exercise was sufficient to downregulate these genes when NASH was present; however, the change in diet combined with exercise (group 5) was not sufficient to decrease AOX1 activity, and enhanced it.

### 3.6 The effects of exercise on the HFD activation of hepatic nuclear receptors

Even though the study design was different from this study, causing some slight divergences in the expression levels, HFD and exercise have been shown to modulate the activity of liver nuclear receptors (Li et al., 2019; Melo et al., 2021). Here we found that HFD increased the expression of all nuclear receptor's tested, and for genes related to aryl hydrocarbon receptor (AHR), constitutive androstane receptor (CAR), and pregnane x receptor (PXR), exercise combined with HFD (group 4), significantly (*p* < 0.05) enhanced its expression when compared to the sedentary HFD group (Figure 7b). CYP3A11, specifically, doubled its expression after exercise in the HFD group. Peroxisome proliferator‐activated receptor α and δ (PPPARα and PPARδ), and CYP4A10 were also significantly (*p* < 0.05) enhanced by exercise along with HFD (Figure 7c). These results agree with previous studies that showed PPPARα’s, PPARδ’s mRNA expression increased with exercise, and PPARδ’s protein expression also increased after 12 weeks of high‐fat followed by exercise in skeletal muscle in rats (Cho et al., 2016; Kannisto et al., 2006).

The present study showed that peroxisome proliferator‐activated receptor γ (PPARγ) and CD36 were significantly (*p* < 0.05) reduced in the group that combined exercise with HFD (group 4) when compared to the sedentary HFD group (Figure 7c). Both these receptors play an essential role in facilitating fat storage (Corrales et al., 2018; Pepino et al., 2014), and our results suggest that exercise can counteract lipid accumulation. Exercise with a control diet decreased the expression of ATP Binding Cassette G5(ABCG5), but significantly (*p* < 0.05) enhanced the expression of ATP Binding Cassette B4 and B11 (ABCB4 and ABCB11) (Figure 7d).

In group 5, where exercise was combined with a change in diet, the liver nuclear receptor's activity was distinct. The combined intervention decreased the expression of all AHR, CAR, PXR, liver X receptor (LXR), and farnesoid X receptor (FXR)‐related genes when compared to group 4 (Exercise and HFD). The same was seen for PPARα, PPARδ, PPARγ, and CYP4A10, but not for ACOX 1 and CD36 (those were significantly (*p* < 0.05) decreased when compared to group 4). This result demonstrates yet again that combining exercise with a change in diet effects gene expression differently than intervening with endurance training alone.

## 4 DISCUSSION

Non‐alcoholic fatty liver disease affects up to one‐third of the population in western countries, and diet along with lack of exercise, genetic predisposition, and metabolic sexual dimorphism is the main causal agents (Li et al., 2019; Melo et al., 2021). Therefore, chronic endurance exercise and change to a healthier diet with lower fat content are therapeutic strategies in the treatment of NAFLD. In this study, we examined the effect of exercise on clinical markers of NAFLD in a mouse liver by human‐relevant rodent models for NAFLD and exercise and confirmed our hypothesis that exercise is able to ameliorate the NAFLD onset; however, only the intervention of exercise along with diet change can revert fatty liver.

Metabolic, cardiorespiratory, and endocrine pathways targeted by chronic endurance exercise have been widely identified in the literature. It was shown before that physical exercise in humans and rodents counteract the different stages of the development of NASH (Bae et al., 2012; Guo et al., 2015; Hallsworth & Trenell, 2020; Nath et al., 2020; Thorp & Stine, 2020; Van der Windt et al., 2018), and exercise positively modulates the murine liver genetic profile through a decrease in pathways related to insulin resistance, steatosis, fibrosis, and inflammation, suggesting a therapeutic strategy for liver diseases (Melo et al., 2021). Nevertheless, the specific liver cellular and molecular pathways modified by exercise remain not unsolved.

The current study uses a human translatable stress‐free exercise endurance protocol and the chronic western diet feeding protocol that approximates the spectrum of liver damages seen in human NAFLD (Li et al., 2018; Stephenson et al., 2018). Our exercise protocol allows mice to run for longer periods in a controlled approach translatable to human dosages of endurance exercise, with a slow increase in velocities to allow the animal to adjust without the use of any adverse stimulus, such as electric shocks or pocking (Melo & Hagar, 2019).

Given that, in this study, we identified the gene expression and structural changes caused by endurance exercise in a NAFLD onset and the potential protective role it has. In addition, we explored the benefits of combining exercise with a change in diet as a treatment for NASH. Exercise itself reduced the size of lipid droplets and the amount of lipid accumulation markers, ameliorated HFD induced hepatic fibrosis and collagen, and reduced HFD hepatic inflammation and oxidative stress. On top of that, exercise intervention along with a change in diet reversed HFD‐induced steatosis in histology analysis, indicating that the complete lifestyle change, change in exercise along with a change in diet, as the best approach for NASH treatment.

At the genetic level, heme oxygenase 1 (HMOX1), an essential gene in the defense against oxidative stress (Chang et al., 2015) and considered one of the most sensitive and reliable indicators of oxidative stress increased with exercise while eating high‐fat chow. Higher HMOX1 activity is correlated with less frequent and less severe NAFLD (Raffaele et al., 2019; Severson et al., 2016), indicating that exercise was a therapeutic approach for NAFLD. On top of that, the upregulation of AOX1 and P47PHOX suggests oxidative damage (Li et al., 2019), and exercise was sufficient to downregulate these genes when NASH was present; however, the change in diet combined with exercise (group 5) was not sufficient to decrease AOX1 activity and actually enhanced it.

The genes related to inflammatory cytokines that play critical roles in the progression of NAFLD, Tnf, Il‐6, and Ifn, were upregulated with HFD. The upregulation of these genes suggests an increase in hepatic stellate cell proliferation after high‐fat diet exposure and exercise (Li et al., 2019; Van der Windt et al., 2018). Il‐6 expression, specifically, increased significantly with exercise for both control diet and high‐fat diet, but decreased in the combined intervention of change in diet plus exercise. This suggests that the combination of change in diet and training decreases hepatic stellate cell recruitment in accordance with a previous study also in C57BL/6 mice (Cho et al., 2016). Given that our results suggest that exercise ameliorates liver inflammatory response that is induced by pre‐existing fatty liver.

Additionally, COL1A1, TIMP1, and TGF‐Β, genes related to fibrosis in the liver, significantly increased in HFD groups (groups 3, 4, and 5) and were downregulated after exercise in the high‐fat diet groups, and both COL1A1 and TIMP1 genes were significantly downregulated after exercise in the control diet groups, in conformity with previous studies (Cho et al., 2016; Takahashi & Fukusato, 2014). Moreover, a comprehensive change in lifestyle (diet combined with exercise) had an additive effect in the expression of all three COL1A1, TIMP1, and TGF‐Β genes when compared to group 4, in which mice gradually endurance‐trained but continued with HFD.

Vegf, a key pro‐angiogenic regulator gene increased with high‐fat diet exposure, however, decreased significantly with exercise in accordance with the literature (Fang & Tang, 2017), with and without change in diet. Angiogenesis is a key event in the progression of NAFLD (Lefere et al., 2020), and VEGF, supported by the activation of hypoxia‐inducible factors, is a key pro‐angiogenic regulator of this process. In biopsy‐proven steatosis in patients, serum VEGF levels are higher than in healthy individuals (Coulon et al., 2012, 2013). Consequently, the modulation of Vegf by the high‐fat diet and exercise supports exercise as a good therapeutical tool for NAFLD. Both PPARγ and CD36 genes were significantly reduced with exercise while eating a high‐fat diet. These two genes play an essential role in facilitating fat storage (Corrales et al., 2018; Pepino et al., 2014), thus our gene expression results confirm that the exercise protocol implemented here can counteract lipid accumulation.

When endurance training is combined with a change in diet, CCND1 gene expression was augmented. An increase in CCND1 expression was previously shown to be involved in tissue regeneration, suggesting exercise and change in diet as a better tissue regenerator (Brett et al., 2020). In accordance with the literature (Hao et al., 2010; Nadeau et al., 2006), the genes involved in cholesterol synthesis and lipid transportation, Srebf1 and Srebf2 and Cpt1A increased its expression with high‐fat diet exposure alone, and when the change in diet was combined with exercise, it was sufficient to decrease its expressions. Moreover, endurance training had an additive effect on the induction of these genes while exposed to high‐fat diet, which is not in accordance with the literature (Ghareghani et al., 2018). However, the exercise methodology used in the study performed by Ghareghani et al, the forced treadmill, is a different methodology than the one used in this study and is known for not being human translatable and cause stress to the animals (Melo & Hagar, 2019).

One limitation in this study is that a variation in NAFLD/NASH severity may be seen in animals within the same group. It may be caused by the difference in daily food consumption since the mice were fed ad libitum. However, the weekly body weight measurements avoid this to happen, and if any bigger changes were seen in one animal of the group, we would separate animals into subgroups with a minimum of three mice per group. Another drawback is that only males are used in this study. The reason for that is that female mice are known to run longer in time and distance (better adaptative range in VO2max and skeletal muscle mass) and were more accepting of the exercise protocol when compared to male mice (Li et al., 2019). Thus, our choice of using male mice is because that gives us the worst‐case scenario for the responses of exercise in the liver. However, female mice need to be investigated under the same circumstances.

We showed here that exercise can amend the NAFLD onset, however, only the intervention in which exercise was accompanied by a change in diet, from high‐fat diet to low‐fat diet, the NAFLD was reverted. Additional investigation is necessary to strengthen the benefits of exercise by itself and exercise with a change in diet in non‐alcoholic liver disease's prevention, progression, and treatment. The next steps would be a further in‐depth pathway analysis and protein level analysis of how exercise and diet affect the liver. Also, more testing using the same methodology but in female mice are needed. Work is underway to fill these gaps.

## CONFLICT OF INTEREST

The authors declare no conflict of interest.

## AUTHOR CONTRIBUTIONS

Luma Melo, Amit Hagar, and James E Klaunig conceived and planned the experiments. Luma Melo and Merve Bilici carried out the experiments and sample preparation. Luma Melo, Amit Hagar, and James E Klaunig contributed to the interpretation of the results. Luma Melo took the lead in writing the manuscript. All authors provided critical feedback and helped shape the research, analysis, and manuscript.

## ETHICAL STATEMENT

The authors consciously assure that for the manuscript that the material is the authors’ own original work, which has not been previously published elsewhere, the paper is not currently being considered for publication elsewhere, the paper reflects the authors’ own research and analysis truthfully and completely, the paper properly credits the meaningful contributions of co‐authors and co‐researchers, the results are appropriately placed in the context of prior and existing research, all sources used are properly disclosed (correct citation), and all authors have been personally and actively involved in substantial work leading to the paper, and will take public responsibility for its content.

## Supporting information



Supplementary Material
