# Supplementary material for: The effect of endurance training on non‐alcoholic fatty liver disease in mice
Source: Physiol Rep. 2021 Aug 2;9(15):e14926. doi: 10.14814/phy2.14926 (PMC8329433; doi:10.14814/phy2.14926)
Supplement: Supplementary file 1 — Supplementary Material [file PHY2-9-e14926-s001.docx]

Supplementary Data

**Supplementary Data 1:** List of primers used for qRT-PCR analysis

Melo, Hagar A, Klaunig JE. Supplementary Table 1. figshare: 2021. <https://figshare.com/articles/dataset/Supplementary_Table_1/13618886>

**Supplementary Data 2:** Image J parameters used for histology slides quantifications

Threshold was the same for all images in this study. Threshold was set as 0-167, default, red, with stack histogram. For counting cell quantity, the background was subtracted, noise was set as despeckle, and image was converted to 8-bit and binary. Particle sizes were set as 120-infinity pixel^2, and circularity of 0 to 1 (including holes). For fat droplets, images were first sharpened and “Find Edges” was used. Then images were converted to 8-bit and followed the same parameters as for counting cell quantity. For quantifying fibrosis, image was set as RGB Stack first and then threshold was set for quantification. The %Area, Area and Std Dev were gathered for analysis.

**Supplementary Data 3:** Whisker plots of all genes analyzed by qRT-PCR, grouped accordingly to their function: fatty acid metabolism, fibrosis, cell proliferation, inflammation, oxidative stress, and hepatic nuclear receptor activation.

| **Fatty Acid Metabolism** | |
| --- | --- |
| 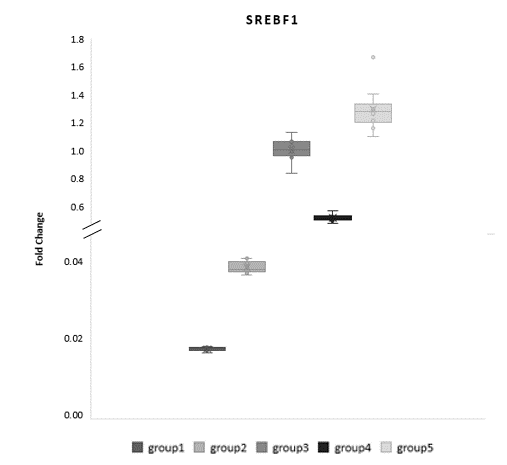 | 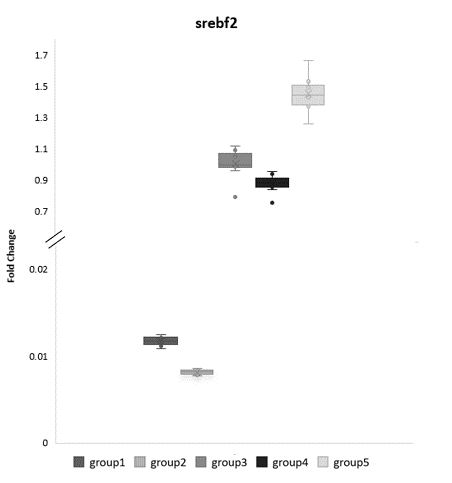 |
| 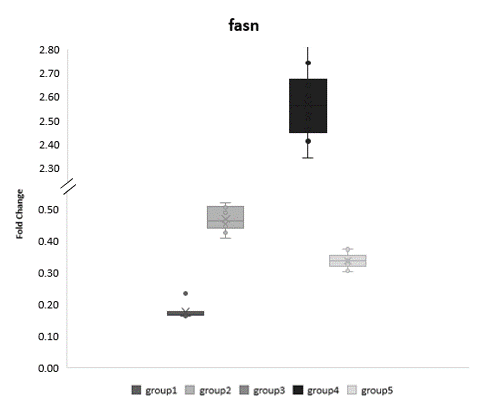 | 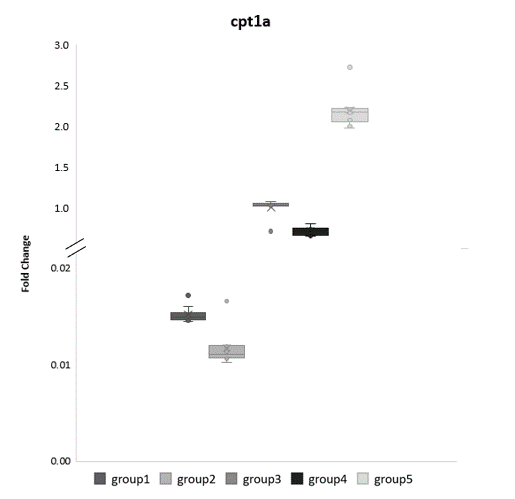 |
| 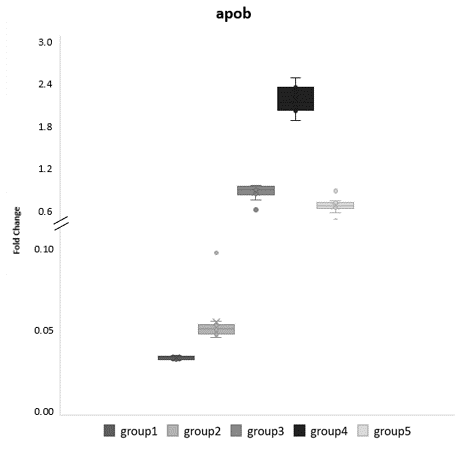 | |

| **Fibrosis** | |
| --- | --- |
| 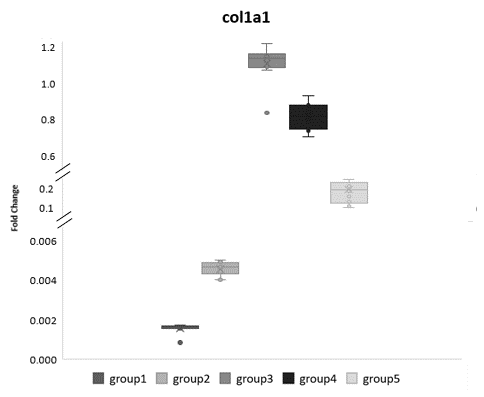 | 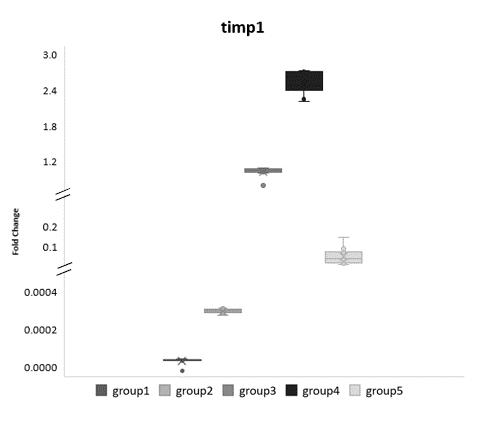 |
| 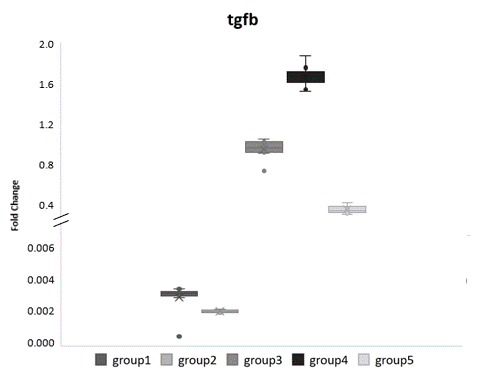 | |

| **Cell proliferation** | |
| --- | --- |
| 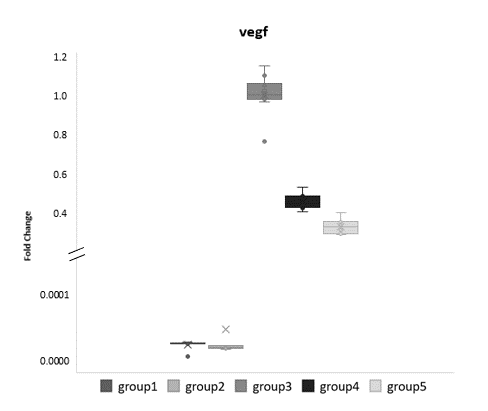 | 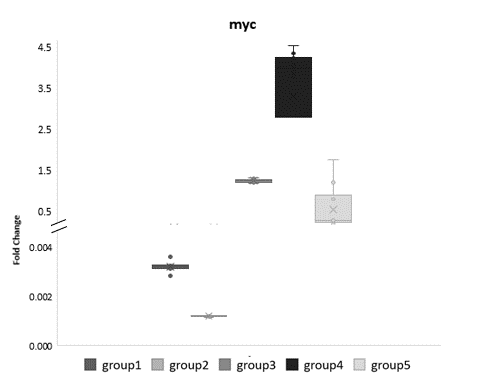 |
| 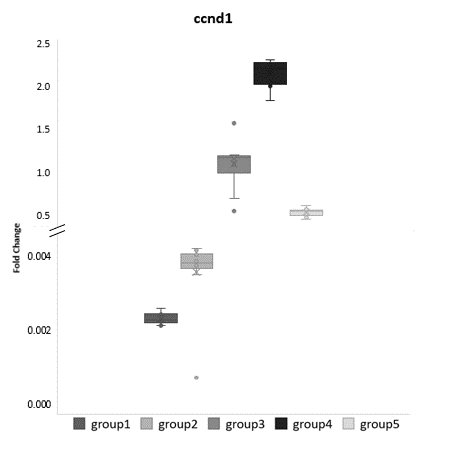 | |

| **Inflammation** | |
| --- | --- |
| 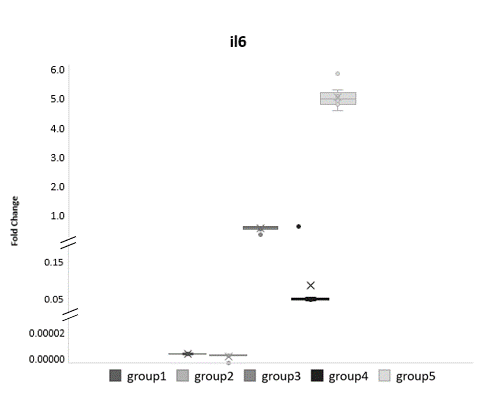 | 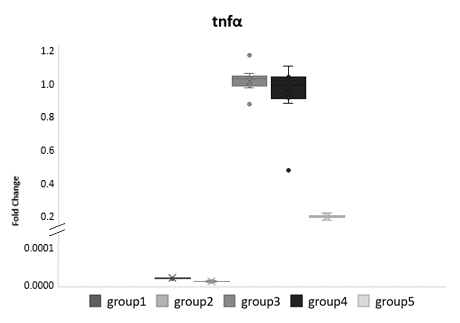 |
| 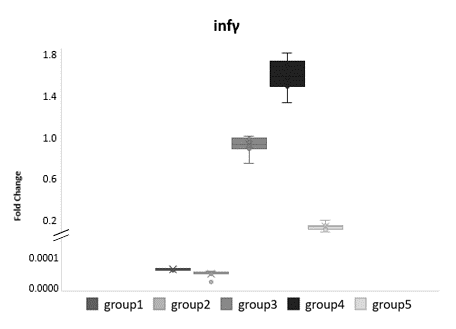 | |

| **Oxidative stress** | |
| --- | --- |
| 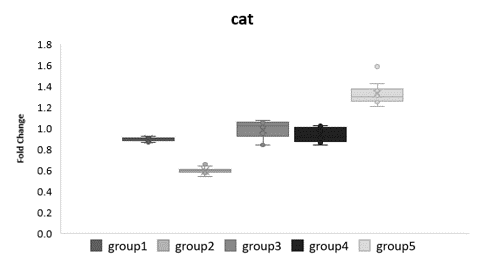 | 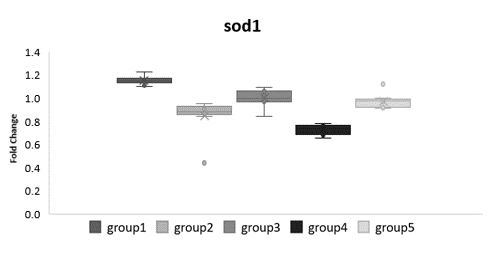 |
| 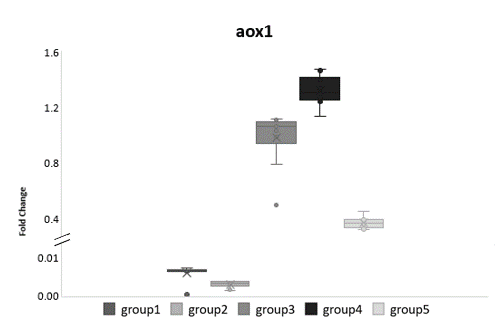 | 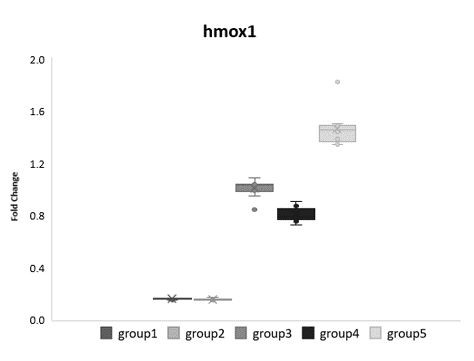 |
| 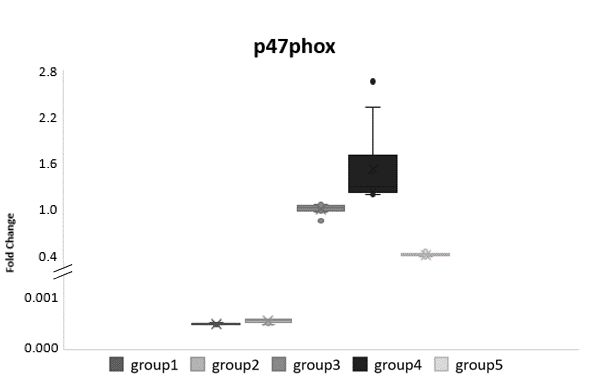 | |

| **Hepatic nuclear receptor activation** | |
| --- | --- |
| 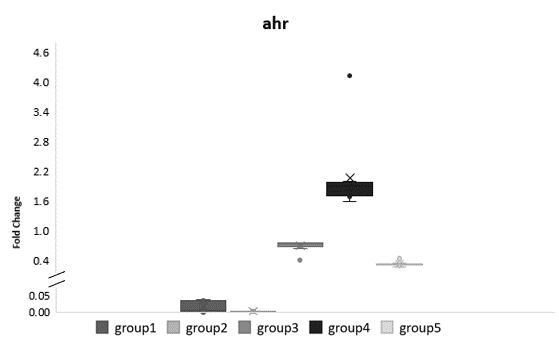 | 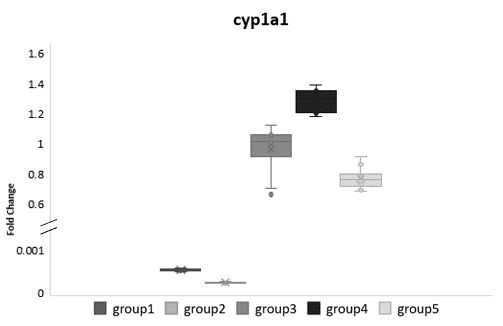 |
| 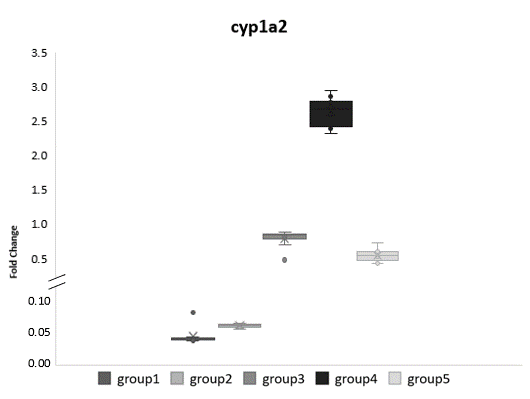 | 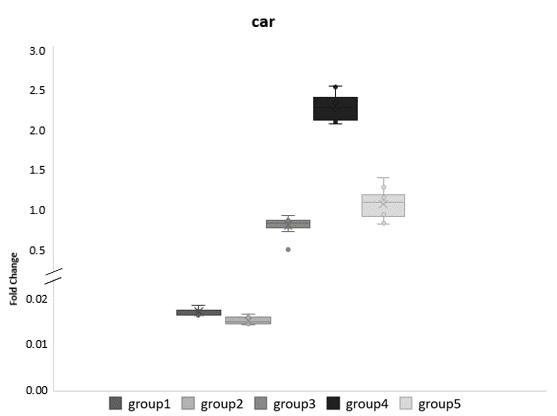 |
| 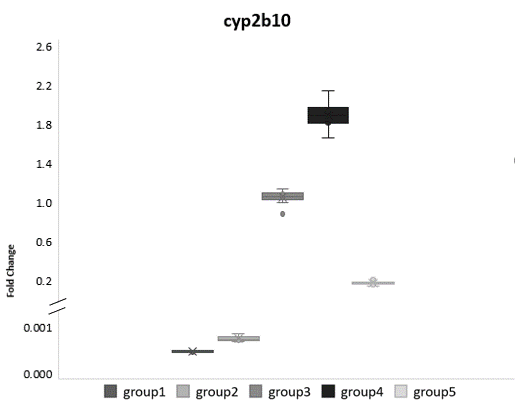 | 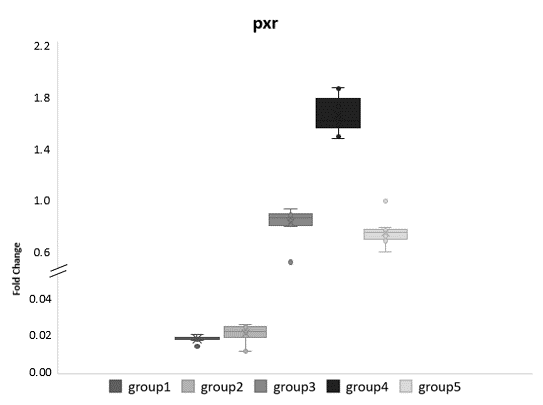 |
| 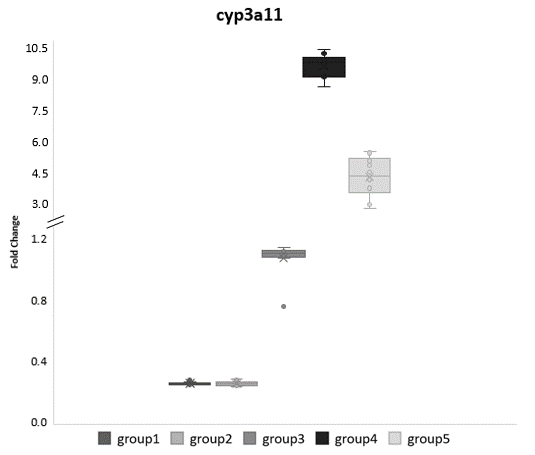 | 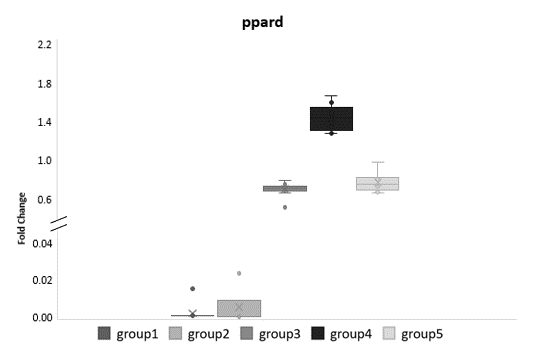 |
| 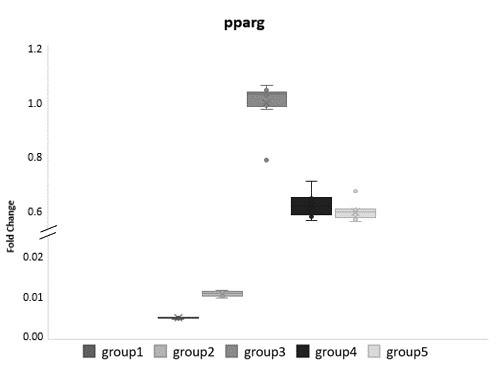 | 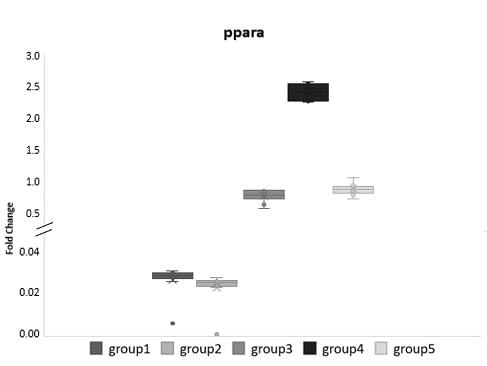 |
| 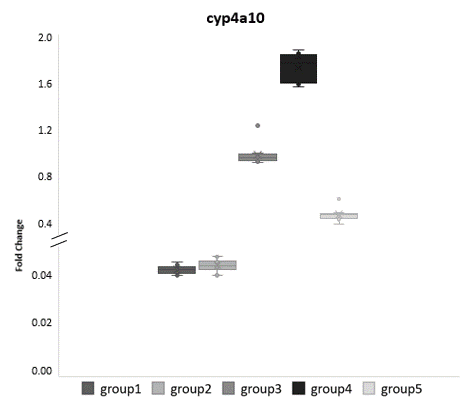 | 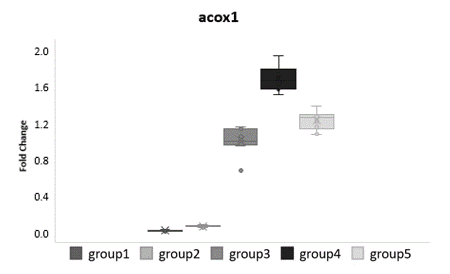 |
| 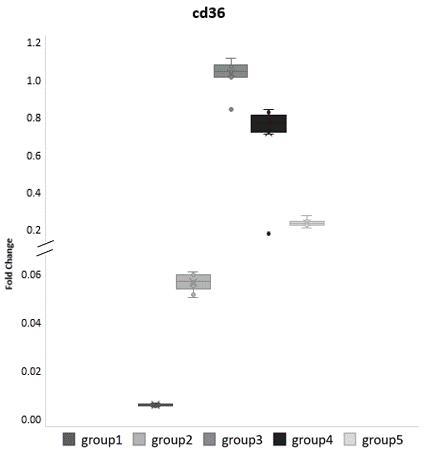 | 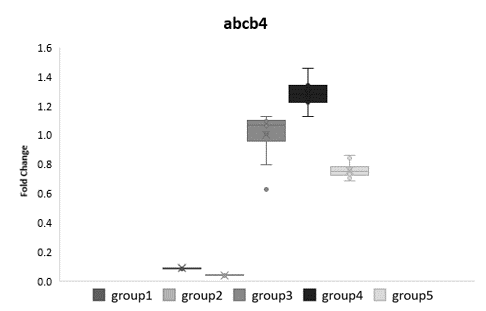 |
| 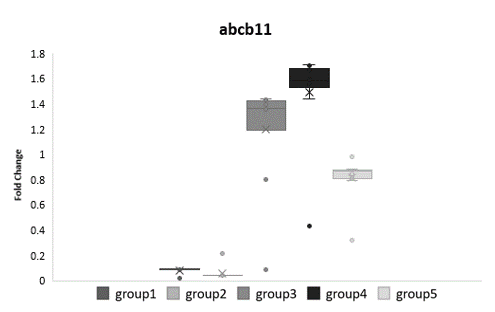 | 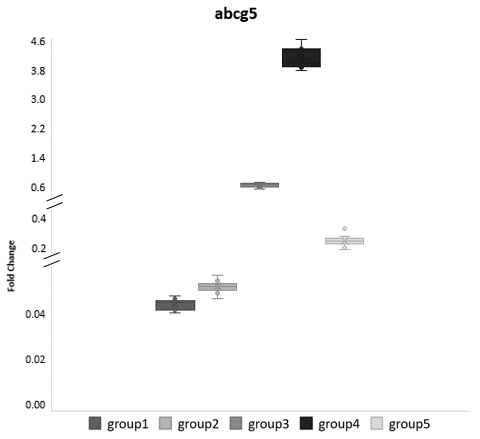 |
| 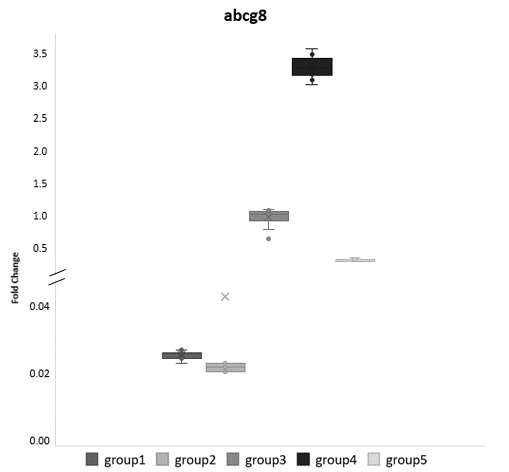 | 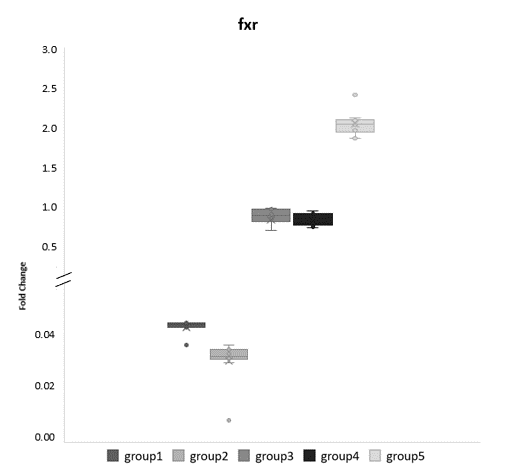 |
| 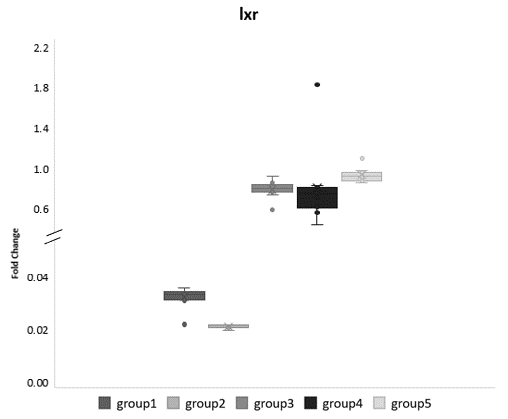 | |

**Supplementary Data 4:** P-values for body weight are provided. The values were calculated for per week of the study. Statistical difference, significantly different when p < 0.05, was determined using either student t-test for two groups or ANOVA when comparing three or more groups. Post-hoc analysis was conducted to investigate changes between groups, if a statistically significant ANOVA result was identified.

**Supplementary Data 5:** P-values for food intake are provided. The values were calculated for per week of the study. Statistical difference, significantly different when p < 0.05, was determined using either student t-test for two groups or ANOVA when comparing three or more groups. Post-hoc analysis was conducted to investigate changes between groups, if a statistically significant ANOVA result was identified.

**Supplementary Data 6:** P-values for liver weight are provided. The values were calculated for data collected in the end of the study. Statistical difference, significantly different when p < 0.05, was determined using either student t-test for two groups or ANOVA when comparing three or more groups. Post-hoc analysis was conducted to investigate changes between groups, if a statistically significant ANOVA result was identified.

|  | **p-value for Liver Weights (LW)** | **p-value for Relative LW** |
| --- | --- | --- |
| **ND+Sed compared to HFD+Sed** | 9.85E-08 | 3.82E-04 |
| **ND+Sed compared to HFD+Ex** | 1.71E-04 | 2.51E-04 |
| **ND+Sed compared to ND+Ex** | 8.02E-01 | 8.16E-01 |
| **ND+Sed compared to HFD/ND+Ex** | 9.78E-01 | 9.28E-01 |
| **ND+Ex compared to HFD+Ex** | 1.79E-04 | 7.04E-05 |
| **ND+Ex compared to HFD/ND+Ex** | 7.82E-01 | 7.69E-01 |
| **HFD+Sed compared to HFD+Ex** | 9.52E-04 | 1.22E-02 |
| **HFD+Sed compared to ND+Ex** | 1.22E-07 | 7.68E-05 |
| **HFD+Sed compared to HFD/ND+Ex** | 1.01E-07 | 3.85E-04 |
| **HFD+Ex compared to HFD/ND+Ex** | 1.77E-04 | 5.24E-04 |
